# Is the diagnosis and treatment of depression gender-biased? Evidence from a population-based aging cohort in Sweden

**DOI:** 10.1186/s12939-024-02320-2

**Published:** 2024-11-27

**Authors:** Amaia Bacigalupe, Unai Martín, Federico Triolo, Linnea Sjöberg, Therese Rydberg Sterner, Serhiy Dekhtyar, Laura Fratiglioni, Amaia Calderón-Larrañaga

**Affiliations:** 1 Department of Sociology and Social Work. Social Determinants of Health and Demographic Change-OPIK Research Group, University of the Basque Country, UPV/EHU, Leioa, Spain; 2 Aging Research Center, Department of Neurobiology, Care Sciences and Society, Karolinska Institutet and Stockholm University, Stockholm, Sweden; 3 Centre for Aging and Health (AgeCap), The University of Gothenburg, Gothenburg, Sweden; 4 Neuropsychiatric Epidemiology Unit, Department of Psychiatry and Neurochemistry, Institute of Neuroscience and Physiology, Sahlgrenska Academy, The University of Gothenburg, Mölndal, Sweden; 5 Stockholm Gerontology Research Center, Stockholm, Sweden

**Keywords:** Depression, Antidepressant use, Gender inequalities, Social inequalities

## Abstract

**Background:**

As compared to men, older women´s higher rates of depression diagnosis and antidepressant use are widely reported. We aimed to: a) explore whether there is a potential gender bias in the clinical diagnosis of depression and antidepressant prescription in an older population from Stockholm; and 2) analyze if such gender bias differs by patients’ age and socioeconomic status.

**Methods:**

We used data from the Swedish National Study on Aging and Care in Kungsholmen, SNAC-K (*N* = 2,941). We compared gender differences in: (a) clinical diagnosis of depression according to the Swedish National Patient Register (ICD-10 codes F32-F34; F412) (“register-based diagnosis”); (b) SNAC-K-based diagnosis of depression, partially gender-blind, using the Comprehensive Psychopathological Rating Scale (CPRS) and the DSM-IV-TR (“SNAC-K based diagnosis); and (c) antidepressant use (ATC code N06A). To analyze the magnitude of the gender bias in the register-based diagnosis of depression and in antidepressant use, and the role of potential moderating factors, prevalence ratios (PR) were calculated using Poisson regression models. Models were run separately by age and social class.

**Results:**

Women had a 63% higher probability of having a register-based diagnosis of depression (PR = 1.63[1.23–2.15]) and a 79% higher probability of using antidepressants (PR = 1.79[1.34–2.40]). No gender differences were observed in the SNAC-K-based diagnosis of depression. The gender differences in the register-based diagnosis were narrowed, although remained significant, after considering age, depressive symptoms, and health services use (PR = 1.44[1.10–1.88]), as well as the register-based diagnosis in the case of antidepressant use (PR = 1.31[1.04–1.64]). This gender bias was larger among the younger-old and the most advantaged social class.

**Conclusion:**

A gender-bias was identified in the diagnosis and treatment of depression in older adults within the Swedish healthcare setting, which could imply that health services may be contributing to the medicalization of women’s mental health. Gender-sensitive clinical and public health interventions are essential to reduce gender disparities in mental healthcare, also in old age.

## Background

The fact that women suffer from worse mental health than men has been repeatedly reported across the psychiatric epidemiological literature. Most of the studies describe that, in the adult population, women are nearly twice as likely as men to have depression or anxiety [1]. Women also tend to receive more psychotropic drug prescriptions, especially anxiolytics and antidepressants [2]. Among the older population, evidence also shows similar relevant sex differences in rates of depression and in the levels of depressive symptoms, which could even increase in the highest age groups [3]. 

While the causes of depression have been extensively studied, the factors explaining the sex and gender differences in depression have been examined to a lower extent [4], particularly in older adults [5]. Apart from a possible genetic and biological predisposition [6], which has shown a limited capacity to explain cross-country variability [7], other explanations point to structural inequalities that confront women with more adverse life conditions [8]. Moreover, women’s longer life expectancy and higher prevalence of chronic health conditions [9], as well as gender differences in coping styles [10], could also play a role in explaining such a gender gap in the diagnosis of depression. Of special interest to this study is another factor related to a possible gender-biased clinical practice [11], but research in this field is scarce and outdated. According to this hypothesis, physicians would diagnose depression and prescribe antidepressants more frequently in women, even with a similar mental health burden as men [2, 12, 13], especially among the elderly and more socioeconomically disadvantaged populations [4, 14]. This over-detection in women has been related to clinician and patient characteristics, comorbidities, and other aspects of the clinician-patient relationship [12, 15, 16]. Simultaneously, an under-detection of depression in men has also been suggested, since physicians are usually more likely to be aware of or act upon mental health issues manifesting in women [17]. Also, women have more frequent contacts with health services, especially primary care services [18], and may thus be at increased risk of being over-diagnosed with depression and receiving unnecessary drug prescriptions compared to men [15]. 

Sweden is known for having high rates of gender equality and relatively small social inequalities. Nonetheless, differences in depression among older men and women are still present, although they seem to be lower than in other European countries [19]. The population-based Swedish National study on Aging and Care in Kungsholmen (SNAC-K), which this paper is based on, offers a unique way to assess the extent of a potential gender bias in the detection and treatment of depression in the health system. In detail, SNAC-K contains information on depression diagnosis from two different sources: the Swedish National Patient Register (NPR), which includes all inpatient and specialized outpatient healthcare in Sweden, and the diagnosis made by experienced physicians from SNAC-K, which is partially gender-blind.

The present study has two main objectives: (1) to explore whether there is a potential gender bias in depression diagnosis and antidepressant prescription within the health system, in an older population from an urban area in Stockholm; and (2) to analyze if and how such gender bias differs by patients’ age and social class.

## Methods

### Study design and population

We used data from SNAC-K, a longitudinal study of adults 60 years and above, living at home or in an institution in the district of Kungsholmen (Stockholm), Sweden [20]. Data from the baseline examination were collected between 2001 and 2004 (participation rate: 73,3%). Data were gathered through clinical examinations, interviews, questionnaires, and tests conducted by nurses, physicians, and psychologists. Data from SNAC-K were linked with the NPR, which includes information on all inpatient and specialized outpatient care in Sweden.

Respondents with definite or questionable dementia diagnoses were excluded from the analyses to minimize recall bias (*n* = 321). Moreover, all those subjects without information on depression diagnosis, depressive symptoms, or antidepressant use were also excluded (*n* = 101). The final sample consisted of *N* = 2,941. The Regional Ethical Review Board in Stockholm approved all phases of the SNAC-K study, and all participants or their next of kin signed a written informed consent.

### Assessment of depression, antidepressant use and health services use

#### Register-based diagnosis of depression

Physician-made diagnoses of depression and/or mood disorders in the inpatient and/or specialized outpatient health services were retrieved through the NPR, using the International Classification of Diseases 10th Revision (ICD-10-SE) codes F32-F34 and F412 up to five years before the baseline SNAC-K visit [21]. 

#### SNAC-K-based diagnosis of depression

Diagnoses of major or minor depression were derived using an algorithm explained in a previous study [22]. Briefly, depressive symptoms and signs were collected during the clinical examination by trained SNAC-K physicians through the Comprehensive Psychopathological Rating Scale (CPRS), a semi-structured interview designed to evaluate the severity and frequency of psychiatric symptoms. Specific symptoms were then used to derive the nine criteria from the Diagnostic and Statistical Manual of Mental Disorders (DSM), which were applied to ascertain major (≥ 5 five symptoms, with at least one core symptom) and minor (2–4 five symptoms, with at least one core symptom) depression according to the DSM-IV-TR. This part of the procedure was carried out by researchers, independently of physicians’ symptom assessments, and without knowledge of participants’ sex, making it a partially gender-blind proxy for depression diagnosis.

#### Burden of depressive symptoms

The Montgomery-Åsberg Depression Rating Scale (MADRS) was used to rate the severity of depressive symptoms. The MADRS is a 10-item subscale of the CPRS, which ranges from 0 to 60 [22]. The variable was used in its continuous format.

#### Antidepressant use

Medication information was collected during the SNAC-K physician interviews, and each drug was coded according to the Anatomical Therapeutic Chemical (ATC) classification. Antidepressant use was considered upon the presence of any drug within the ATC class N06A.

#### Assessment of health services use

The total number of participants’ visits to inpatient and/or specialized outpatient care during the year before the SNAC-K interview was collected from the NPR.

### Assessment of socio-demographic characteristics

*Social class* was derived from participants’ longest-held occupation, which was classified using the Swedish Socioeconomic Index and categorized into manual and non-manual [23]. *Age* was categorized into two groups: <78 and ≥ 78 years. *Sex* was collected as a binary variable: men and women. However, given our aim to understand how social and cultural gender constructions can influence the observed differences between men and women, the results and discussion sections will be described in terms of gender differences.

### Statistical analyses

We compared gender differences in the crude and age-standardized prevalences of the register-based diagnosis of depression (5-year prevalence), the SNAC-K-based diagnosis of depression (SNAC-K baseline prevalence), antidepressant use (SNAC-K baseline prevalence), as well as the burden of depressive symptoms (SNAC-K baseline means). The total sample was used as the standard population. We also calculated the crude and age-adjusted differences in the prevalences between men and women (and their statistical significance) employing prevalence ratios (PR), using Poisson regression models with robust error variance [24], and taking males as the reference group. As a way to assess potential over- and under-detection of depression and over- and under-prescription of antidepressants, the correspondence between the SNAC-K based diagnosis with both the register-based diagnosis and the use of antidepressants was explored using a cross-tabulation table, including a chi-square test.

To analyze the potential gender bias in the diagnosis of depression within the health system, as well as the role played by potential patient-related demographic and clinical factors, PRs of the register-based diagnosis were calculated for women vs. men, adjusting sequentially for age (continuous), burden of depressive symptoms (continuous MADRS), and health services use (0; 1; 2–4; and ≥ 5 visits). To analyze the potential gender bias in the prescription of antidepressants, the register-based diagnosis of depression was included as an additional adjustment variable. All these models were also run separately by age group and social class.

## Results

At baseline, the 5-year prevalence of register-based depression diagnosis was 6.0% and 9.7% for men and women, respectively. The point prevalence of the SNAC-K-based diagnosis was lower (4.5% and 5.2%, respectively). The mean MADRS score was higher in women. Antidepressant use was recorded in 5.3% of men and 9.8% of women. In general, women were older, visited the health services more frequently and had a lower social class. Detailed characteristics of the sample are reported in Table 1.

**Table 1:** Socio-demographic and health characteristics of the study population (*N* = 2,941)

	Men (*n* = 1,091)		Women (*n* = 1,850)	
	*N*	%	*N*	%
**Register-based diagnosis of depression**				
*No*	1026	94.0	1671	90.3
*Yes*	65	6.0	179	9.7
**SNAC-K-based diagnosis of depression**				
*No*	1042	95.5	1754	94.8
*Yes*	49	4.5	96	5.2
**Burden of depressive symptoms (MADRS)** Mean (SD)	2.26 (3.68)		2.94 (4.13)	
**Use of antidepressants**				
*No*	1033	94.7	1669	90.2
*Yes*	58	5.3	181	9.8
**Age group**				
<78 years	728	66.7	982	53.1
≥78 years	363	33.3	868	46.9
**Physician visits in the last year**				
*0*	222	20.4	197	10.6
*1–2*	219	20.1	383	20.7
*3–5*	210	19.2	369	19.9
*6–10*	154	14.1	358	19.4
*>10*	286	26.2	543	29.4
**Social class**				
*Non-manual*	888	81.4	1404	75.9
*Manual*	180	16.5	352	19.0
*Missing*	23	2.1	94	5.1

As shown in Table 2, women had a 63% higher probability of having a register-based diagnosis of depression (PR = 1.63 [1.23–2.16]) and a 79% higher probability of receiving antidepressants (PR = 1.79 [1.34–2.40]). No gender differences were observed in the SNAC-K-based diagnosis of depression (PR = 1.06 [0.75–1.50]). Of note, the burden of depressive symptoms among those who had a SNAC-K based diagnosis was higher in women than in men (age-standardized mean value of MADRS = 14.7 and 12.4 respectively; *p* = 0.0342; data not shown in tables/figure).

**Table 2:** Age-standardized prevalence (%) of depression-related variables by sex, and age-adjusted prevalence ratios (PR)* in women (reference: men)

	Men (%)	Women (%)	Age-adjusted PR (95% CI)
**SNAC-K-based diagnosis of depression**	5.0	5.1	1.06 (0.75–1.50)
**Register-based diagnosis of depression**	6.1	9.7	**1.63 (1.23–2.16)**
**Use of antidepressants**	5.6	9.8	**1.79 (1.34–2.40)**

*In bold, statistically significant PRs (*p* value ≤ 0.05)

The correspondence between the SNAC-K based diagnosis, the register-based diagnosis, and the use of antidepressants is shown in Table 3. Among men without a SNAC-K-based diagnosis of depression, 4.6% were labelled as depressed in the register data, while this percentage increased to 7.8% in women (*p* value 0.001). Potential over-diagnosis of depression was, thus, significantly higher among women. On the contrary, among participants with a SNAC-K-based diagnosis of depression, 65.3% of men and 56.3% of women did not have a register-based diagnosis, indicating that the potential under-diagnosis of depression was higher among men, even if differences were not statistically significant (*p* value = 0.294). Moreover, while a significantly higher proportion of women without a SNAC-K diagnosis of depression were using antidepressants compared to men (8.4% versus 4.8%, respectively, *p* value 0.000), a significantly lower proportion of men diagnosed as depressed in SNAC-K were using them compared to women (16.3% versus 34.4%, respectively, *p* value 0.022).

**Table 3:** Prevalence (%) of register-based diagnosis of depression and antidepressant use in men and women stratified by having or not a SNAC-K-based diagnosis of depression

		Register-based diagnosis of depression			Antidepressant use		
		No	Yes	*p* value	No	Yes	*p* value
**Not diagnosed as depressed according to SNAC-K**	Men	95.4	4.6	0.001	95.2	4.8	0.000
	Women	92.2	7.8		91.6	8.4	
**Diagnosed as depressed according to SNAC-K**	Men	65.3	34.7	0.294	83.7	16.3	0.022
	Women	56.3	43.8		65.6	34.4	

Table 4 presents the PRs of a register-based diagnosis of depression in women compared to men, according to different adjustments. The gender differences in the register-based diagnosis persisted, even after considering participants’ age, burden of depressive symptoms, and frequency of health services use, with a prevalence 44% higher in women (PR = 1.44 [1.10–1.88]) after adjusting for all covariates. Among these factors, differences in the burden of depressive symptoms accounted for the largest part of the gender gap in the register-based diagnosis.

**Table 4:** Prevalence ratios (PR)* of register-based diagnosis of depression in women (reference: men) in the total population and stratified by age group and social class

	Model I: Crude PR	Model II Age-adjusted PR	Model III: model II + MADRS	Model IV: model III + health services use
Total	**1.62 (1.24–2.14)**	**1.63 (1.23–2.16)**	**1.48 (1.13–1.93)**	**1.44 (1.10–1.88)**
**Age group**				
*<78 years*	**1.91 (1.33–2.75)**	**1.93 (1.34–2.78)**	**1.84 (1.31–2.59)**	**1.75 (1.24–2.47)**
*≥78 years*	1.25 (0.83–1.91)	1.25 (0.82–1.90)	1.09 (0.72–1.64)	1.09 (0.73–1.63)
**Social class**				
*Non-manual*	**1.84 (1.33–2.55)**	**1.86 (1.34–2.58)**	**1.72 (1.25–2.35)**	**1.65 (1.20–2.27)**
*Manual*	0.93 (0.53–1.64)	0.95 (0.52–1.74)	0.84 (0.47–1.51)	0.83 (0.47–1.48)

*In bold, statistically significant PRs (*p* value ≤ 0.05)

**Fig. 1:**
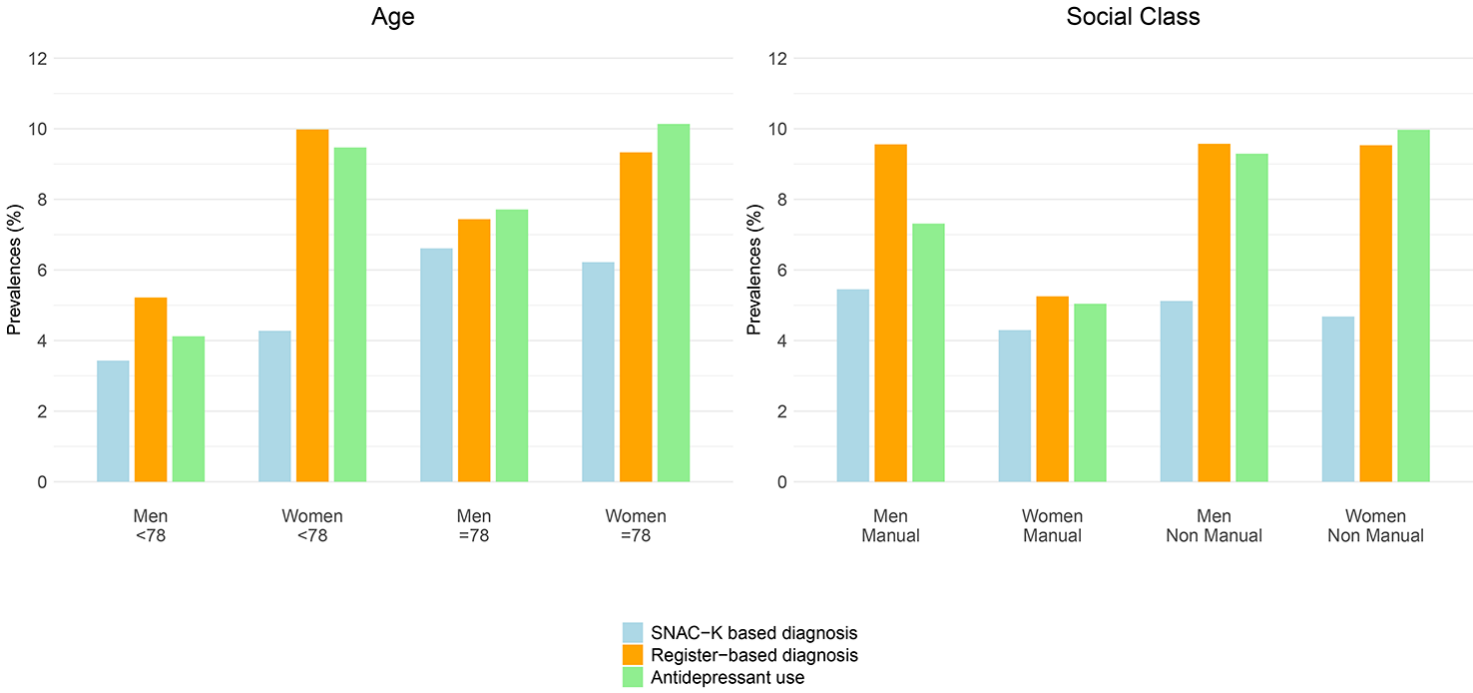
Age-standardized prevalence (%) of register-based and SNAC-K-based diagnosis of depression in men and women, by age group and social class

Women´s increased probability of having a register-based diagnosis of depression was more evident among the younger-old (PR = 1.75 [1.24–2.47]) and in the non-manual social class (PR = 1.65 [1.20–2.27]). Accordingly, it was among younger women where the biggest differences between the prevalence of the register-based and SNAC-K-based diagnoses of depression were found, while men showed smaller differences between both types of diagnoses across age groups (Fig. 1). Regarding social class, men of non-manual social class showed the smallest differences between both types of diagnoses (Fig. 1).

Table 5 describes the PRs of antidepressant use in women versus men, according to different adjustments. Overall, women showed an 84% higher risk of using antidepressants (PR = 1.84 [1.38–2.45]). Also, when continuous age, the burden of depressive symptoms, the register-based diagnosis of depression, and health services use were considered, the association remained significant (PR = 1.31 [1.04–1.64]). Among these factors, differences in the register-based diagnosis of depression between men and women explained most of the gender gap in antidepressant use. Again, the gender difference was higher among the younger-old (PR = 1.42 [1.04–1.94]) and the non-manual social class (PR = 1.34 [1.03–1.73]).

**Table 5:** Prevalence ratio (PR)* of antidepressant use in women (reference: men) in the total population and stratified by age group and social class

	Model I: Crude	Model II Age-adjusted	Model III: model II + MADRS	Model IV: model III + register-based diagnosis of depression	Model V: model IV + health services use
Total	**1.84 (1.38–2.45)**	**1.79 (1.34–2.40)**	**1.66 (1.25–2.22)**	**1.30 (1.04–1.61)**	**1.31 (1.04–1.64)**
**Age group**					
*<78 years*	**2.30 (1.54–3.43)**	**2.30 (1.54–3.44)**	**2.18(1.47–3.24)**	**1.44 (1.06–1.97)**	**1.42 (1.04–1.94)**
*≥78 years*	1.31 (0.87–1.98)	1.31 (0.87–1.96)	1.20 (0.80–1.80)	1.13 (0.84–1.54)	1.13 (0.81–1.59)
**Social class**					
*Non-manual*	**2.04 (1.47–2.85)**	**2.01 (1.44–2.81)**	**1.88 (1.35–2.61)**	**1.33 (1.03–1.72)**	**1.34 (1.03–1.73)**
*Manual*	1.30 (0.70–2.40)	1.21 (0.63–2.32)	1.12 (0.59–2.16)	1.31 (0.85–2.03)	1.27 (0.74–2.15)

*In bold, statistically significant PRs (*p* value ≤ 0.05)

## Discussion

In this large community-based cohort study of Swedish older adults aged ≥ 60 years, the frequency of a register-based diagnosis of depression was significantly higher among women, while no gender differences were observed in the SNAC-K-based (i.e. partially gender-blind) diagnosis of depression. The use of antidepressants was also significantly more frequent among women. These results would imply that a gender-biased diagnosis and treatment of depression was ascertained. Indeed, women had a 44% higher probability of having a register-based diagnosis and a 31% higher probability of receiving treatment for depression than men, given the same age, burden of depressive symptoms, and frequency of health services use. Moreover, this gender bias seemed to be larger among the younger-old and the more advantaged social class. Differences in the burden of depressive symptoms contributed the most to explaining the gender gap in the register-based diagnosis of depression. In the case of the use of antidepressants, differences in the clinical diagnosis of depression between men and women accounted for the majority of the observed gap.

This study extends previous research in two main ways. First, it uses two different measures of depression diagnosis; a variable reflecting physician performance (the register-based diagnosis) and a proxy of actual depression status (SNAC-K-based diagnosis). Given that these two diagnostic measures are rarely available for the same sample, studies on gender differences in the diagnosis of depression have tended to use other indicators of mental health to operationalize depression status, such as the MHI-5, GHQ, or BDI scales [4, 12–15, 17], which have a different meaning from a medical diagnosis. Second, the SNAC-K-based diagnosis of depression is almost gender-blind, given that the final assignment of the diagnosis is made without knowing the sex of the participant, allowing for a more accurate assessment of real differences in depression prevalence between men and women. Indeed, when using this diagnostic measure, results showed no differences between men and women.  

Our finding of the gender differences in the register-based prevalence of depression is consistent with the literature, which indicates that older women have on average 2.2 times higher rates compared to men [25]. A review on depression in older age also found a significantly greater likelihood of a depression diagnosis based on clinical interviews or cut-off scores in women as compared to men [5]. However, the lack of gender differences according to the SNAC-K diagnosis was somewhat unexpected [26], probably due to the fact that there are hardly any studies that apply gender-neutral assessments of depression, as done herein. In community-based studies using non-clinical measures of mental health, negligible differences between men and women are usually found, and women may even score higher across the domains of life satisfaction, happiness, and/or well-being than men [27]. A previous study using SNAC-K data on self-reported life satisfaction and positive and negative affect neither found any gender differences [28], which further supports our hypothesis that differences in depression diagnosis and treatment between men and women may partly stem from a gender-biased healthcare provision.

According to the literature, the reasons behind this gender-biased clinical practice can be diverse and interconnected. Regarding clinician-related factors, previous studies have shown that physicians assessed differently identical patient vignettes that only varied according to gender, judging female patients as more emotional and plaintive [16, 29]. This could be the result of a *double standard of mental health* in men and women [30], which pathologizes the feminine normative identity more easily. The reason behind this could be that, while male personality traits are typically judged as inducing good mental health, female traits may be understood as symptoms of impared mental health. The few previous studies looking at the effects of gender on the diagnosis of depression within health services suggest an over-diagnosis in women, given the same number and severity of depressive symptoms and equal healthcare use among men and women [15], in line with the results of the present study. Women tend to use antidepressants more frequently than men across different European countries, given equal depressive status assessed through the DSM-IV classification [31]. Of note, male prescribers are more likely than female professionals to prescribe benzodiazepines to female patients compared to male patients [32, 33]. These trends may also be more evident if the prescription is made by mental health specialists rather than GPs [12]. Simultaneously to a potential over-diagnosis in women, our work suggested an under-detection of depression in men (despite the lack of statistical significance), which may be because physicians are usually more likely to be aware of or act upon women’s mental health issues [12, 17]. Men also find it more difficult to express their emotional distress because such an attitude may challenge conventional notions of masculinity, and they could, therefore, trivialize their symptoms during the medical consultation, making it more difficult for professionals to diagnose depression [34]. In our study, men diagnosed as depressed in SNAC-K were also less likely to receive antidepressant treatment, which may be because certain side effects of this medication (on sexual life for example) may be taken into greater consideration in male patients [35].

Moreover, diagnostic and mental health screening tools could also be gender biased, as they may favor the expression of female over male symptoms of depression, by focusing on internalizing symptoms (e.g. sadness, loss of interest or pleasure in previously-enjoyed activities) rather than those symptoms that are more sensitive to masculine role norms (e.g. anger, substance misuse, emotion suppression, risk-taking) [36]. According to a meta-analysis, depressed women reported symptoms that are included as diagnostic criteria in the DSM-V or ICD-10 at a higher frequency and intensity than men [37].  Still, other studies on the effect of this potential measurement bias have been inconclusive [38]. In our study, we could assume that this bias was small, as no differences between men and women were observed concerning the SNAC-K-based depression diagnosis, but the situation may be different in the clinical setting. Patient-related factors may also contribute to gender biases in the diagnostic assessment of depression. Qualitative evidence on experiences of depression in early late life shows that old men find it harder to discuss their emotions than women since they believe that expressing sadness, crying, and other depressive symptoms is not as socially acceptable for men as for women [39]. Indeed, gender differences in the frequency and type of symptom reporting due to masculinity/femininity constructions have been previously reported [40]. 

To our knowledge, few studies have analyzed whether a gender bias in the diagnosis and treatment of depression is dependent on social position, indicating an increased gender bias among lower socioeconomic positions [4, 14]. On the contrary, our work showed greater gender differences in the higher social class, as both types of depression diagnoses were most concordant among men of higher social class compared to any other group (including women of any social class and men in the manual class). The theory of intersectionality stresses that an advantaged position in terms of gender and social class may play a protective role against biased clinical practices that can lead to the medicalization of mental health [41], which could partially explain our results. A more in-depth analysis is, however, required.

This study has several strengths, including its population-based design, large sample size, high participation rate, the use of multiple clinical measures to compare depression diagnosis, and linkage to inpatient and specialized outpatient national registers. However, the study also has specific limitations. First, the sample came from a comparatively well-off and homogenous area in Stockholm, so a more diverse population in terms of socioeconomic status would likely have enabled more nuanced intersectional analyses of social inequalities. Second, the register-based depression diagnosis was based on ICD codes while the SNAC-K diagnosis used DSM codes, which could capture different subpopulations of older people with depression. Both measures also relied on different time-frames, so comparisons should be made with caution. This could have an impact on the analyses of the over- and under-diagnosis of depression, as the register-based diagnosis went up to five years before the baseline SNAC-K visit, and subjects could be in remission by the time the SNAC-K visit took place. However, there is no clear evidence on potential gender differences in the rates of remission of depressive symptoms [42], which reduces the likelihood of such a bias. Third, the register-based diagnosis did not include data from primary healthcare, which could under-estimate the observed gender differences, as women have more frequent contact with primary care services. Fourth, even if the SNAC-K-based diagnosis of depression could be considered as being gender-blind given that the final diagnostic assignment was made without knowing the sex of the participant, we cannot assure that the initial assessment of the CPRS items by SNAC-K physicians was not gender-biased. These two last limitations would in any case further reinforce our findings. Finally, we did not have information about antidepressant indications for SNAC-K participants, so we cannot rule out that they were prescribed for other different conditions such as anxiety, which is very frequently comorbid with depression, particularly in women. However, anxiety symptoms are already accounted for in the MADRS scale, so we may have partially controlled for this potential bias in our analyses.

Our findings have important implications for a gender sensitive public-health practice since they suggest that there is a gender bias in the diagnosis and treatment of depression in older adults, which could be leading to the medicalization of women´s mental health alongside an under-detection/treatment of depression in men. The androcentric construction of biomedicine and its translation to clinical practice, but also the gender mandates conditioning the expressiveness of symptoms and the unequal expectations on the performance of healthcare professionals faced with male or female patients, are key to understanding the triggers of this gender bias.

## Conclusion

Older women have higher rates of diagnosed depression in the inpatient and specialized outpatient settings and use antidepressants more frequently than men. Gender-biased clinical practice may potentially contribute to these disparities, with physicians possibly diagnosing depression and prescribing antidepressants to women more often than men, despite similar mental health needs. Indeed, this study identifies a gender bias in the diagnosis and treatment of depression within the Swedish healthcare setting, whereby women are 44% more likely to receive a diagnosis and 31% more likely to use antidepressants than men, even when age, burden of depressive symptoms, and healthcare use are accounted for. Moreover, this bias is more pronounced among younger-olds and subjects in higher social positions. Simultaneously to a potential over-diagnosis/treatment in women, an under-detection/treatment of depression in men could also be happening, although to a lesser extent. Our findings advocate for gender-sensitive clinical and public health interventions to reduce gender disparities in mental healthcare, also in old age.

## Data Availability

Data, analytic methods, and materials are available to other researchers for replication purposes. The study reported in the manuscript was not pre-registered. Data are from the SNAC-K project, a population-based study on aging and dementia (http://www.snac-k.se/). Access to these original data is available to the research community upon approval by the SNAC-K data management and maintenance committee. Applications for accessing these data can be submitted to Maria Wahlberg (Maria.Wahlberg@ki.se) at the Aging Research Center, Karolinska Institutet.

## Abbreviations

ATC: Anatomical Therapeutic Chemical
CPRS: Comprehensive Psychopathological Rating Scale
ICD-10: International Classification of Diseases 10th Revision
MADRS: Montgomery-Åsberg Depression Rating Scale
NPR: National Patient Register
PR: Prevalence Ratio
SNAC-K: Swedish National study on Aging and Care in Kungsholmen

## References

[1] Riecher-Rössler A. Sex and gender differences in mental disorders. Lancet Psychiatry. 2017;4(1):8–9. 10.1016/S2215-0366(16)30348-0 27856397

[2] Boyd A, Van de Velde S, Pivette M, ten Have M, Florescu S, O’Neill S, et al. Gender differences in psychotropic use across Europe: results from a large cross-sectional, population-based study. Eur Psychiatry. 2015;30:778–88. 26052073 10.1016/j.eurpsy.2015.05.001

[3] Kiely KM, Brady B, Byles J. Gender, mental health and ageing. Maturitas. 2019;129:76–84. 10.1016/j.maturitas.2019.09.004 31547918

[4] Cabezas-Rodríguez A, Bacigalupe A, Martín U. Diagnosis and treatment of depression in Spain: are there gender inequalities? Int J Environ Res Public Health. 2020;17:1–10. 10.3390/ijerph17249232 PMC7763221 33321853

[5] Girgus JS, Yang K, Ferri CV. The gender difference in depression: are elderly women at greater risk for depression than elderly men? Geriatrics (Basel). 2017;2(4):35. 10.3390/geriatrics2040035 PMC6371140 31011045

[6] Labaka A, Goñi-Balentziaga O, Lebeña A, Pérez-Tejada J. Biological Sex differences in Depression: a systematic review. Biol Res Nurs. 2018;20:383–92. 29759000 10.1177/1099800418776082

[7] Van de Velde S, Bracke P, Levecque K. Gender differences in depression in 23 European countries. Cross-national variation in the gender gap in depression. Soc Sci Med. 2010;71:305–13. 20483518 10.1016/j.socscimed.2010.03.035

[8] Van de Velde S, Huijts T, Bracke P, Bambra C. Macro-level gender equality and depression in men and women in Europe. Sociol Health Illn. 2013;35:682–98. 23145770 10.1111/j.1467-9566.2012.01521.x

[9] Noh JW, Kwon YD, Park J, Oh IH, Kim J. Relationship between physical disability and depression by gender: a panel regression model. PLoS One. 2016;11(11):e0166238. 10.1371/journal.pone.0166238 PMC5130183 27902709

[10] Lutzky SM, Knight BG. Explaining gender differences in caregiver distress: the roles of emotional attentiveness and coping styles. Psychol Aging. 1994;9(4):513–9. 10.1037//0882-7974.9.4.513 7893422

[11] Fitzgerald C, Hurst S. Implicit bias in healthcare professionals: a systematic review. BMC Med Ethics. 2017;18(1):19. 10.1186/s12910-017-0179-8 PMC5333436 28249596

[12] Potts MK, Audrey Burnam M, Wells KB, Aiken L, Cohen A, Keherer B, et al. Gender differences in Depression detection: a comparison of Clinician diagnosis and standardized Assessment. Psychol Assess. 1991;3(4),609–615.

[13] Redman S, Webb GR, Hennrikus DJ, Gordon JJ, Sanson-Fisher RW. The effects of gender on diagnosis of psychological disturbance. J Behav Med. 1991;14(5):527–40. 10.1007/BF00845109 1744914

[14] Bacigalupe A, Martín U. Gender inequalities in depression/anxiety and the consumption of psychotropic drugs: are we medicalising women’s mental health? Scand J Public Health. 2021;49:317–24. 32755295 10.1177/1403494820944736

[15] Bertakis KD, Helms LJ, Callahan EJ, Azari R, Leigh P, Robbins JA. Patient gender differences in the diagnosis of depression in primary care. J Womens Health Gend Based Med. 2001;10(7):689–98. 10.1089/15246090152563579 11571099

[16] Stoppe G, Sandholzer H, Huppertz C, Duwe H, Staedt J. Gender differences in the recognition of depression in old age. Maturitas. 1999;32(3):205–12. 10.1016/s0378-5122(99)00024-9 10515678

[17] Borowsky SJ, Rubenstein LV, Meredith LS, Camp P, Jackson-Triche M, Wells KB. Who is at Risk of Nondetection of Mental Health Problems in Primary Care? J Gen Intern Med. 2000;15:381–8. 10886472 10.1046/j.1525-1497.2000.12088.x PMC1495467

[18] Ballering AV, Olde Hartman TC, Verheij R, Rosmalen JGM. Sex and gender differences in primary care help-seeking for common somatic symptoms: a longitudinal study. Scand J Prim Health Care. 2023;41:132–9. 36995265 10.1080/02813432.2023.2191653 PMC10193899

[19] Schmitz A, Brandt M. Gendered patterns of depression and its determinants in older europeans. Arch Gerontol Geriatr. 2019;82:207–16. 30831527 10.1016/j.archger.2019.02.015

[20] Lagergren M, Fratiglioini L, Hallberg IR, Bergllund J, Elmstahl S, Hagbeg B, et al. A longitudinal study integrating population, care and social services data. The Swedish National study on aging and care (SNAC). Aging Clin Exp Res. 2004;16:158–68. 15195992 10.1007/BF03324546

[21] Calderón-Larrañaga A, Vetrano DL, Onder G, Gimeno-Feliu LA, Coscollar-Santaliestra C, Carfí A, et al. Assessing and measuring chronic multimorbidity in the older Population: a proposal for its operationalization. J Gerontol A Biol Sci Med Sci. 2017;72(10):1417–23. 10.1093/gerona/glw233 PMC5861938 28003375

[22] Sjöberg L, Karlsson B, Atti AR, Skoog I, Fratiglioni L, Wang HX. Prevalence of depression: comparisons of different depression definitions in population-based samples of older adults. J Affect Disord. 2017;221:123–31. 28645024 10.1016/j.jad.2017.06.011

[23] Harber-Aschan L, Calderón-Larrañaga A, Darin-Mattson A, Hu X, Fratiglioni L, Dekhtyar S. Beyond the social gradient: the role of lifelong socioeconomic status in older adults’ health trajectories. Aging. 2020;12:24693–708. 33349620 10.18632/aging.202342 PMC7803509

[24] Zou G. A modified Poisson Regression Approach to prospective studies with Binary Data. Am J Epidemiol. 2004;159:702–6. 15033648 10.1093/aje/kwh090

[25] Salk RH, Hyde JS, Abramson LY. Gender differences in depression in representative national samples: Meta-analyses of diagnoses and symptoms. Psychol Bull. 2017;143:783–822. 28447828 10.1037/bul0000102 PMC5532074

[26] Wang K, Lu H, Cheung EFC, Neumann DL, Shum DHK, Chan RCK. Female preponderance’ of depression in non-clinical populations: a meta-analytic study. Front Psychol. 2016;15:7:1398. 10.3389/fpsyg.2016.01398 PMC5023676 27695433

[27] Pinquart M, Sörensen S. Gender differences in self-concept and psychological well-being in old age: a meta-analysis. J Gerontol B Psychol Sci Soc Sci. 2001;56(4):P195–213. 10.1093/geronb/56.4.p195 11445606

[28] Saadeh M, Welmer AK, Dekhtyar S, Fratiglioni L, Calderón-Larrañaga A. The role of psychological and social well-being on physical function trajectories in older adults. J Gerontol A Biol Sci Med Sci. 2020;75:1579–85. 10.1093/gerona/glaa114 PMC7357580 32384140

[29] Colameco S, Becker LA, Simpson M, Jersey N. Sex Bias in the Assessment of Patient complaints. J Fam Pract. 1983;16:1117–21. 6854242

[30] Chesler P. Women and madness. Chicago: Chicago Review; 2018.

[31] Alonso J, Angermeyer MC, Bernert S, Bruffaerts R, Brugha TS, Bryson H, et al. Psychotropic drug utilization in Europe: results from the European study of the Epidemiology of Mental disorders (ESEMeD) project. Acta Psychiatrica Scand Supplement. 2004;109:55–64. 10.1111/j.1600-0047.2004.00331.x 15128388

[32] McIntyre RS, Chen VCH, Lee Y, Lui LMW, Majeed A, Subramaniapillai M, et al. The influence of prescriber and patient gender on the prescription of benzodiazepines: evidence for stereotypes and biases? Soc Psychiatry Psychiatr Epidemiol. 2021;56:1083–9. 33258001 10.1007/s00127-020-01989-4

[33] Bjørner T, Lærum E. Factors associated with high prescribing of benzodiazepines and minor opiates: a survey among general practitioners in Norway. Scand J Prim Health Care. 2003;21:115–20. 12877376 10.1080/02813430310001734

[34] O’Brien R, Hunt K, Hart G. It’s caveman stuff, but that is to a certain extent how guys still operate’: men’s accounts of masculinity and help seeking. Soc Sci Med. 2005;61:503–16. 15899311 10.1016/j.socscimed.2004.12.008

[35] Bacigalupe A, González-Rábago Y, Jiménez-Carrillo M. Gender inequality and mental health medicalization: sociocultural determining factors from the analysis of expert perceptions. Aten Primaria. 2022;54(7):102378. 10.1016/j.aprim.2022.102378 PMC9160668 35653856

[36] Martin LA, Neighbors HW, Griffith DM. The experience of symptoms of depression in men vs women: analysis of the national comorbidity survey replication. JAMA Psychiatry. 2013;70:1100–6. 23986338 10.1001/jamapsychiatry.2013.1985

[37] Cavanagh A, Wilson CJ, Kavanagh DJ, Caputi P. Differences in the expression of symptoms in men Versus Women with Depression: a systematic review and Meta-analysis. Harv Rev Psychiatry. 2017;25:29–38. 28059934 10.1097/HRP.0000000000000128

[38] Jane JS, Oltmanns TF, South SC, Turkheimer E. Gender bias in diagnostic criteria for personality disorders: an item response theory analysis. J Abnorm Psychol. 2007;116:166–75. 17324027 10.1037/0021-843X.116.1.166 PMC4372614

[39] Rydberg Sterner T, Dahlin-Ivanoff S, Gudmundsson P, Wiktorsson S, Hed S, Falk H, et al. ‘I wanted to talk about it, but I couldn’t’, an H70 focus group study about experiencing depression in early late life. BMC Geriatr. 2020;20(1):528. 10.1186/s12877-020-01908-x PMC7720563 33287708

[40] Luppa M, Sikorski C, Luck T, Ehreke L, Konnopka A, Wiese B, et al. Age- and gender-specific prevalence of depression in latest-life - systematic review and meta-analysis. J Affect Disord. 2012;136(3):212–21. 10.1016/j.jad.2010.11.033 21194754

[41] Trygg NF, Gustafsson PE, Månsdotter A. Languishing in the crossroad? A scoping review of intersectional inequalities in mental health. Int J Equity Health. 2019;24;18(1):115. 10.1186/s12939-019-1012-4 PMC6657170 31340832

[42] Entsuah AR, Huang H, Thase ME. Response and remission rates in different subpopulations with Major Depressive Disorder Administered Venlafaxine, selective serotonin reuptake inhibitors, or Placebo. J Clin Psychiatry. 2001;62:869–77. 11775046 10.4088/jcp.v62n1106

